# Antimicrobial Peptide Combination Can Hinder Resistance Evolution

**DOI:** 10.1128/spectrum.00973-22

**Published:** 2022-07-13

**Authors:** Bar Maron, Jens Rolff, Jonathan Friedman, Zvi Hayouka

**Affiliations:** a Institute of Biochemistry, Food Science and Nutrition, The Hebrew University of Jerusalem, Rehovot, Israel; b Department of Plant Pathology and Microbiology, The Hebrew University of Jerusalem, Rehovot, Israel; c Institute of Biology, Evolutionary Biology, Freie University, Berlin, Germany; Agricultural Research Organization, Volcani Center

**Keywords:** experimental evolution, antibiotic resistance, antimicrobial combinations, antimicrobial peptides

## Abstract

Antibiotic-resistant microbial pathogens are becoming a major threat to human health. Therefore, there is an urgent need to develop new alternatives to conventional antibiotics. One such promising alternative is antimicrobial peptides (AMPs), which are produced by virtually all organisms and typically inhibit bacteria via membrane disruption. However, previous studies demonstrated that bacteria can rapidly develop AMP resistance. Here, we study whether combination therapy, known to be able to inhibit the evolution of resistance to conventional antibiotics, can also hinder the evolution of AMP resistance. To do so, we evolved the opportunistic pathogen Staphylococcus aureus in the presence of individual AMPs, AMP pairs, and a combinatorial antimicrobial peptide library. Treatment with some AMP pairs indeed hindered the evolution of resistance compared with individual AMPs. In particular, resistance to pairs was delayed when resistance to the individual AMPs came at a cost of impaired bacterial growth and did not confer cross-resistance to other tested AMPs. The lowest level of resistance evolved during treatment with the combinatorial antimicrobial peptide library termed random antimicrobial peptide mixture, which contains more than a million different peptides. A better understanding of how AMP combinations affect the evolution of resistance is a crucial step in order to design “resistant proof” AMP cocktails that will offer a sustainable treatment option for antibiotic-resistant pathogens.

**IMPORTANCE** The main insights gleaned from this study are the following. (i) AMP combination treatment can delay the evolution of resistance in S. aureus. Treatment with some AMP pairs resulted in significantly lower resistance then treatment with either of the individual AMPs. Treatment with a random AMP library resulted in no detectable resistance. (ii) The rate at which resistance to combination arises correlates with the cost of resistance to individual AMPs and their cross-resistance. In particular, combinations to which the least resistance arose involved AMPs with high fitness cost of resistance and low cross-resistance. (iii) No broad-range AMP resistance evolved. Strains that evolved resistance to some AMPs typically remained sensitive to other AMPs, alleviating concerns regarding the evolution of resistance to immune system AMPs in response to AMP treatment.

## INTRODUCTION

Overuse of antibiotics in both medicine and agriculture has led to antibiotic-resistant microorganisms becoming widespread in environmental and clinical settings. In particular, bacterial pathogens have become a major threat to public health, being responsible for more than 2.8 million infections and more than 35,000 deaths annually in the United States alone (1). Staphylococcus aureus is a significant human pathogen that causes multiple types of infections leading to morbidity and mortality (2, 3). It is known for its exceptional ability to develop resistance toward a multitude of antimicrobials (4).

Several approaches aiming at curbing the rise and spread of resistance have been proposed as follows: (i) prudent use of antimicrobials, (ii) development of new antimicrobials, and (iii) development of treatment strategies that prevent or delay the evolution of antimicrobial resistance. Here, we combine two of these approaches and study whether a treatment strategy based on combinations of antimicrobial peptides (AMPs) can hinder resistance development.

Antimicrobial peptides are a diverse family of compounds produced by virtually all organisms (5–7) that typically inhibit bacteria via several mechanisms, mainly by disrupting bacterial membranes (8–12). AMPs are considered to be a promising novel alternative to traditional antibiotics (13). However, widespread application of AMPs may also cause the rise of AMP-resistant pathogens. Rapid evolution of resistance to AMPs will negate their efficacy and may compromise the activity of AMPs that are part of the immune system. Therefore, there is a need to develop treatment strategies involving AMPs that delay the evolution of resistance to these antimicrobial agents.

Recent studies have shown that resistance to AMPs can evolve in the lab and in nature (14, 15). Several *in vitro* studies showed that AMPs can select for resistant bacteria (16). Perron et al. evolved Escherichia coli and Pseudomonas fluorescens in the presence of pexiganan, a synthetic analogue of frog antimicrobial peptides (magainins) (17). Both bacterial species evolved resistance to pexiganan within approximately 650 generations. In addition, Dobson et al. evolved the Gram-positive bacterium Staphylococcus aureus in the presence of different antimicrobials, including antimicrobial peptides, and showed that S. aureus evolved resistance against AMPs, albeit slower than against antibiotics (18). A lower rate of evolution was also observed against the prokaryotic AMP colistin compared with that against antibiotics (19).

Typically, AMPs are found in diverse mixtures of different peptides or other types of antimicrobial agents rather than individual ones (20–25). Previous studies have shown that different interactions between antimicrobials can affect the evolution of resistance (26, 27). Interestingly, it has been found that AMPs can interact synergistically in pairs and even better in a three-AMP combination (28). Nevertheless, only few studies have been investigating the evolution of resistance toward AMP combinations (18, 29). Dobson et al. found that S. aureus went extinct more rapidly when treated with a mixture of two antimicrobial peptides—pexiganan and melittin—compared to single AMP treatments (18). However, it is still not clear how general these results are and which AMP combinations will lead to delayed resistance evolution.

In this study, we aim to elucidate the evolution of resistance toward individual AMPs and their combinations and the factors that influence the combination’s efficiency. To do so, we have performed experimental evolution of S. aureus in the presence of several individual AMPs and AMP combinations. A better understanding of how AMP combinations affect the evolution of resistance is a crucial step in the development of “resistant proof” AMP cocktails that can offer a sustainable treatment option of antibiotic-resistant pathogens (30).

## RESULTS

### Resistance toward single AMPs evolved readily.

**FIG 1:**
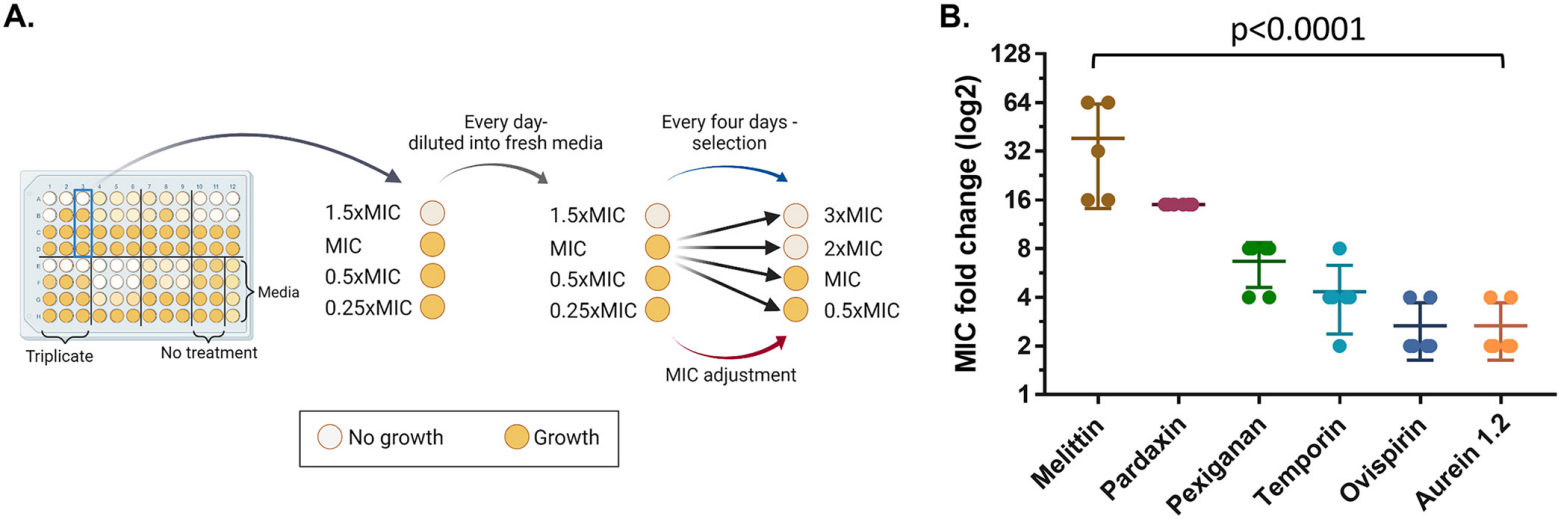
Resistance evolution varied between AMPs. (A) Experimental evolution procedure. Every day, 10 μL of the previous day’s well was copied into a new plate with fresh medium and AMP/antibiotics. Every 4 days, bacteria that grew in the highest AMP concentration were selected and transferred into the four new concentrations of the line (black arrows). AMP/antibiotic concentrations doubled when growth was observed in 4 out of 6 lines in MIC or above. Each 96-well plate contained the following: triplicate of each treatment (in two separate plates, total of 6 lines), 8 wells of bacteria without treatment, and medium control. The experiment continued for approximately 130 generations (29 days). Evolved strains were isolated after 29 days. (B) Resistance of evolved strains. Resistance determined by MIC assay of each strain toward the corresponding AMP. Results shown as fold change of ancestor MIC values (*n* = 6; *n*_mel_ = 5; bars represent mean ± standard deviation [SD]). Significant mean differences determined using Kruskal-Wallis test (see Table S4 in the supplemental material).

First, we performed experimental evolution of S. aureus in the presence of six individual AMPs with different modes of bacterial membrane disruption (see Table S1 in the supplemental material). Prior to the experimental evolution, the antimicrobial activity of each AMP toward S. aureus was evaluated by measuring its MIC (Table S1). The evolution experiment was designed to maintain strong selection for resistance yet to avoid extinction. Therefore, each of six replicate lines of bacteria was exposed to four concentrations of each AMP as follows: 1.5×MIC, 1×MIC, 0.5×MIC, and 0.25×MIC (Fig. 1A). Every four transfers, the MIC was doubled if four out of six lines grew in the MIC or higher. Before the selection, we inoculated the bacteria with AMP-free medium (Mueller-Hinton broth [MHB]) in order to habituate it to the experimental conditions. The bacteria were transferred each day to fresh medium (diluted 1:20) for 7 days. The first experimental evolution assay was performed with six individual AMPs for 29 days (≈130 generations), and each AMP had six parallel independent lines of bacteria.

At the end of the experiment, the evolved strains were isolated, and the MIC values were determined in order to evaluate the level of resistance under the same conditions for all strains. The level of resistance that evolved varied significantly across AMPs (Fig. 1B, *P* < 0.0001; see also Table S4 in the supplemental material). The strains that evolved with melittin had the largest increase in resistance, with a mean MIC that was 38-fold higher than the ancestor strain. The MICs of pexiganan- and temporin-evolved strains increased by 5- and 4-fold, respectively. Interestingly, ovispirin and aurein, for which the MICs increased the least (2-fold on average), both have a carpet model mechanism of action (31, 32) as opposed to the other AMPs.

### Combinations of AMPs can hinder the evolution of resistance.

To investigate the evolution of resistance with AMP combinations, we performed experimental evolution with the the following AMPs for which medium-high resistance evolved: melittin, pexiganan, and temporin (pardaxin AMP was excluded due to solubility issues). Before we started the evolution experiment with AMP combinations, we evaluated the interaction between AMPs using a checkerboard assay. We found that there is no notable synergistic/antagonistic interaction between the tested combinations (see Table S3 in the supplemental material). Therefore, the initial MIC of each combination contained 0.5× MIC of each AMP. The experimental evolution procedure was performed similar to that of the first experiment (approximately 130 generations).

**FIG 2:**
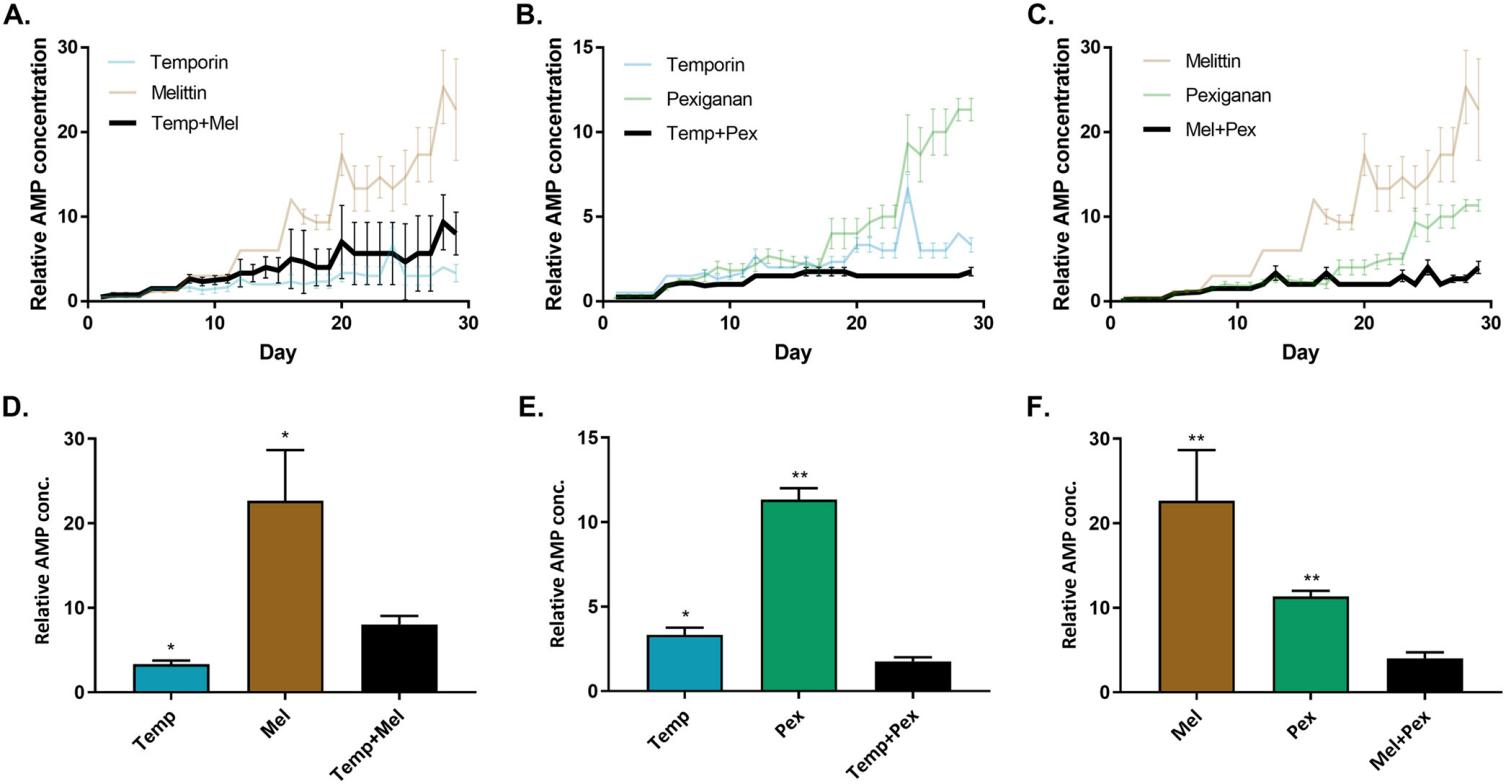
AMP combinations can hinder the evolution of resistance. Data represents the concentration of AMPs where bacteria grew, normalized to initial MIC (*n* = 6; mean ± standard error [SE]; growth defined as OD_595_ > 0.3). (A) Temporin-melittin combination. (B) Temporin-pexiganan combination. (C) Melittin-pexiganan combination. Each graph shows the concentrations of individual AMPs and their combinations through the evolution. (D to F) Comparison of the relative AMP concentration after 29 transfers. Each bar represents the mean of six lines + standard error of the mean (SEM). *, *P* < 0.05; **, *P* < 0.01 (Mann-Whitney U test with Bonferroni correction; results compared to combinations strains). Data of individual AMPs is identical in all panels. Mel, melittin; Pex, pexiganan; Temp, temporin. For additional statistics information, see Table S4 in the supplemental material.

Resistance evolution toward individual AMPs in this experiment was consistent with the results of the first experiment (Fig. 2). Temporin-evolved strains exhibit medium-low resistance, and melittin-evolved strains had relatively high resistance. For the combination treatments, temporin-melittin could not inhibit the evolution of resistance better than temporin alone (Fig. 2A). However, the combination of temporin-pexiganan hindered the evolution of resistance compared with each AMP alone (Fig. 2B). The combination of pexiganan-melittin showed an even stronger “synergistic” effect, where the evolved bacteria managed to grow in an AMP concentration only 4-fold the initial MIC, whereas they could grow in concentrations 10- to 20-fold higher the initial MIC when evolved with the individual AMPs (Fig. 2C). Overall, treatment with some AMP combinations resulted in drastically lower resistance than treatment with each individual compound.

**FIG 3:**
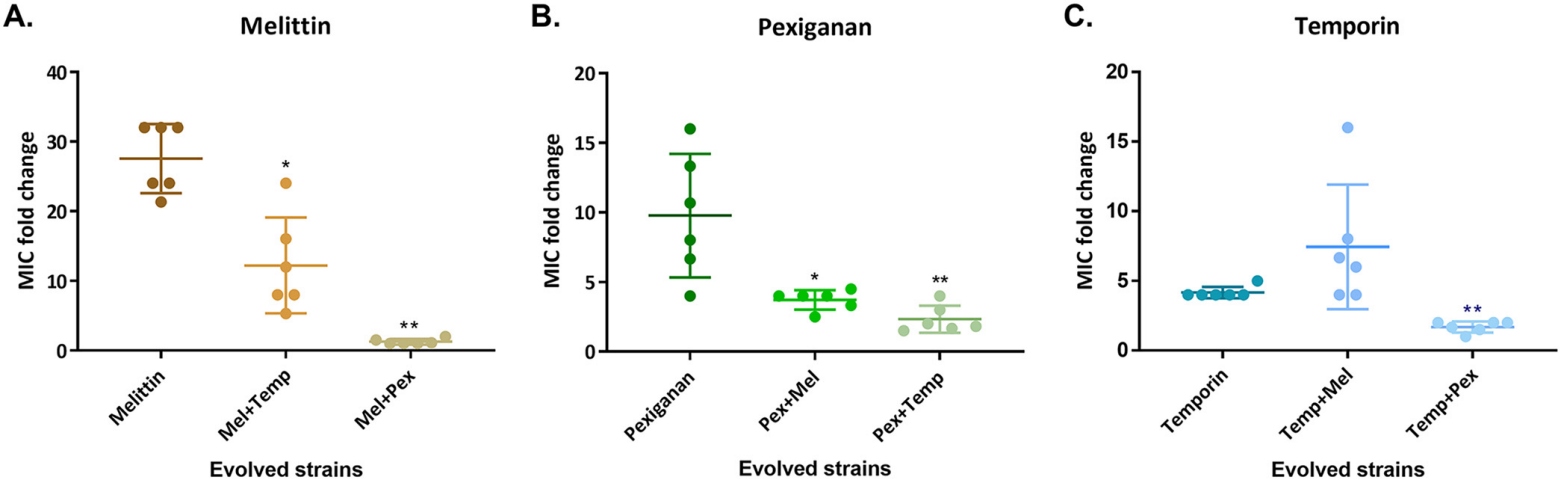
Resistance toward melittin and pexiganan was significantly reduced in most combinations. Each strain isolated from the end of the experiment and MIC values were determine using a standard assay. (A) Melittin-evolved combinations. (B) Pexiganan-evolved combinations. (C) Temporin-evolved combinations. The MIC values refer to the AMP in the title and are normalized to ancestor MIC. Each dot represents a line (*n* = 6), and bars represent mean ± SD. *, *P* < 0.05; **, *P* < 0.01 (Mann-Whitney U test with Bonferroni correction; comparison of each combination to the individual AMP’s strain). Mel, melittin; Pex, pexiganan; Temp, temporin. The results of each dot represent the mean of at least three independent experiments. For additional statistics information, see Table S4 in the supplemental material.

To further verify our findings, we isolated strains from the last day of the experiment and performed a standard MIC assay. The results of this MIC assay are consistent with the resistance levels found during the experimental evolution (Fig. 3). Additionally, the sensitivity of the strain that evolved without AMPs (NPSA) remain similar to the ancestor's (see Table S2 in the supplemental material). All of the combinations that contained melittin were found to reduce the resistance toward it. Pexiganan combinations result in the most effective combinations, as the MIC value toward pexiganan drops from an average of 9-fold change to 3-fold change in the combinations. The results from temporin-evolved strains confirm that these combinations were less effective than temporin alone, except for the pexiganan-temporin combination.

### Resistance to melittin and pexiganan incurred a notable fitness cost.

**FIG 4:**
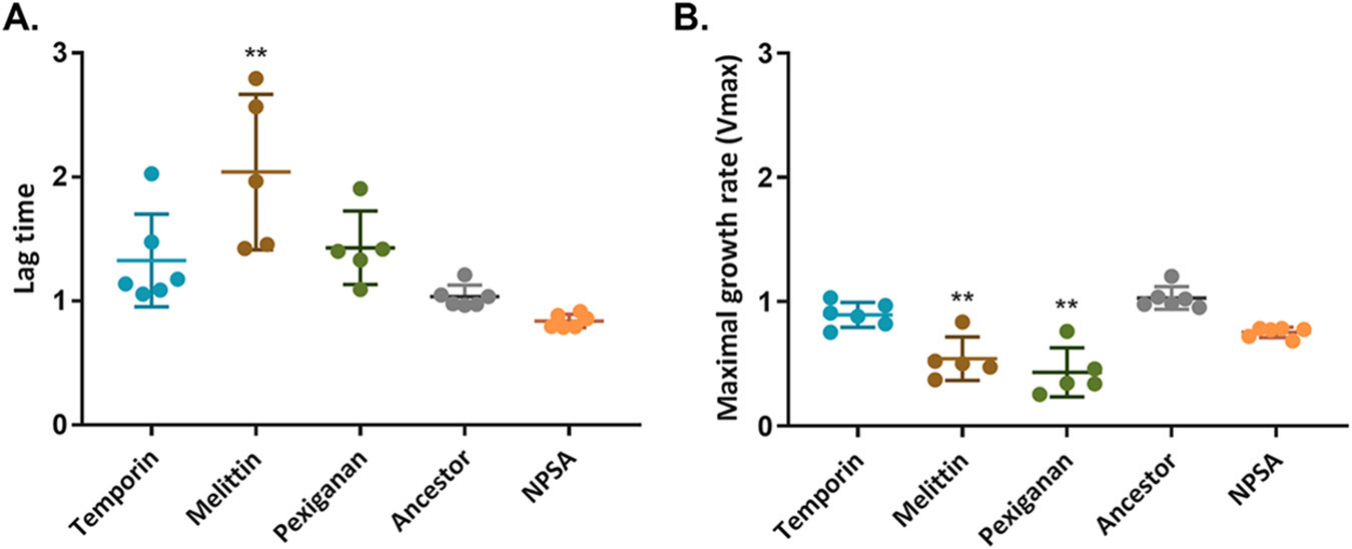
Resistance to melittin and pexiganan incurs a fitness cost in the form of a longer lag time and reduced growth rate. Determination of fitness cost performed by growing the bacteria in the absence of AMPs. OD_595_ was measured every 15 min through 24 h. (A) Lag time. (B) Maximal growth rate (*V*_max_). Values were calculated by plate reader software (Gen 5 and normalized to ancestor strain values). Each dot represents mean of triplicate, bars represent mean ± SD (*n*_melittin, pexiganan_ = 5, Dunn's multiple comparison in reference to the ancestor strain; **, *P* < 0.01). NPSA, evolved strain without AMPs. The results represent three independent experiments. For additional statistics information, see Table S4 in the supplemental material.

Next, we wanted to understand why some combinations are more effective than others in inhibiting the resistance occurrence. One hypothesis is that if there is a significant fitness cost of resistance to individual AMPs, the combination of two high-cost AMPs will better hinder the evolution of resistance. To test this hypothesis, we measured the growth ability of each evolved strain in the absence of AMPs (Fig. 4).

The lag time increased significantly in melittin strains (Fig. 4A) (*P* = 0.0078), and a similar increase was found in the time to achieve maximal growth rate (see Fig. S2 in the supplemental material). Lag time also increased in temporin and pexiganan strains but not statistically significantly (*P*_Temp_ = 0.6585; *P*_Pex_ = 0.3323). Another important aspect of bacterial fitness is the maximal growth rate, which was reduced in both pexiganan and melittin strains but not in temporin-evolved strains (Fig. 4B). The overall fitness, which can be expressed by the area under the growth curve, was significantly attenuated in pexiganan-evolved strains (*P* = 0.0095) and melittin-evolved strains (*P* = 0.05) (Fig. S2). The fitness cost of the strains that evolved with AMP combinations was not impaired, except for temporin-melittin maximal growth rate (see Fig. S3 in the supplemental material). Overall, these results indicate that the AMPs for which resistance incurred the largest fitness costs, melittin and pexiganan, are indeed the ones whose combination most considerably hindered the evolution of resistance.

### Cross-resistance evolved but not toward all AMPs.

**FIG 5:**
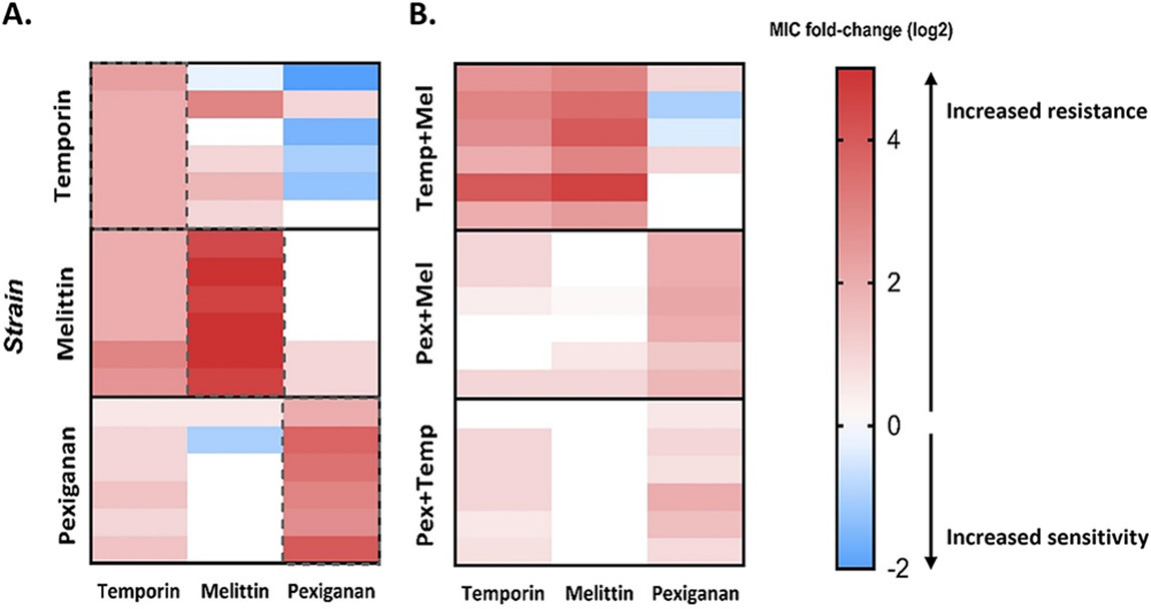
Cross-resistance evolved frequently toward temporin but less toward pexiganan. A standard MIC assay was performed in order to evaluate the cross-resistance and collateral sensitivity. In each experiment, the evolved strain was exposed to different AMPs, with the selected AMP as control. The MIC values present as fold change (log_2_) of the ancestor’s MIC. The results represent the mean of three independent experiments. (A) Individual AMP evolved strains. (B) Two-AMP evolved strains. Red color indicates resistance, and blue indicates sensitivity. Dashed lines indicate the MIC toward the AMP that it evolved with (not cross-resistance or sensitivity).

Another possible mechanism affecting the evolution of resistance to combination therapy is cross-resistance or collateral sensitivity. If resistance toward a single AMP confers resistance to another AMP (cross-resistance), we hypothesize that a combination of these AMPS will be less effective at delaying the evolution of resistance. However, when resistance confers collateral sensitivity toward another AMP, we expect that this combination will be more effective. To examine this hypothesis, we determined the MIC value of each of the evolved strains toward the other AMPs it has not evolved with (Fig. 5). We found that cross-resistance toward temporin was common (Fig. 5A), which may explain why it was not effective at delaying resistance in combination treatments. Cross-resistance to pexiganan evolved significantly less often than to temporin (*P* = 0.0003) (Table S4), and temporin-evolved strains even showed increased sensitivity to pexiganan. These results are consistent with the fact that AMP combinations that contain pexiganan were more effective for delaying resistance evolution. Notably, melittin- and pexiganan-evolved strains were the only AMP pair where no major cross-resistance occurred, which may be another factor contributing to it being the combination for which resistance was most drastically reduced.

To further explore our hypothesis, we have also assayed the resistance of strains evolved with AMP combinations to all three individual AMPs (Fig. 5B). Strains that evolved with a melittin-temporin combination exhibited medium resistance, corresponding to the individual AMPs in Fig. 5A. The strains that evolved in combination with pexiganan presented resistance toward pexiganan but less toward melittin, even when evolved with a melittin-pexiganan combination. Overall, except for 3 strains out of 18, bacteria remained susceptible to at least one AMP, which suggests that resistance toward some AMPs does not confer a general cross-resistance toward other AMPs.

### S. aureus developed low resistance to a combinatorial antimicrobial peptide library.

**FIG 6:**
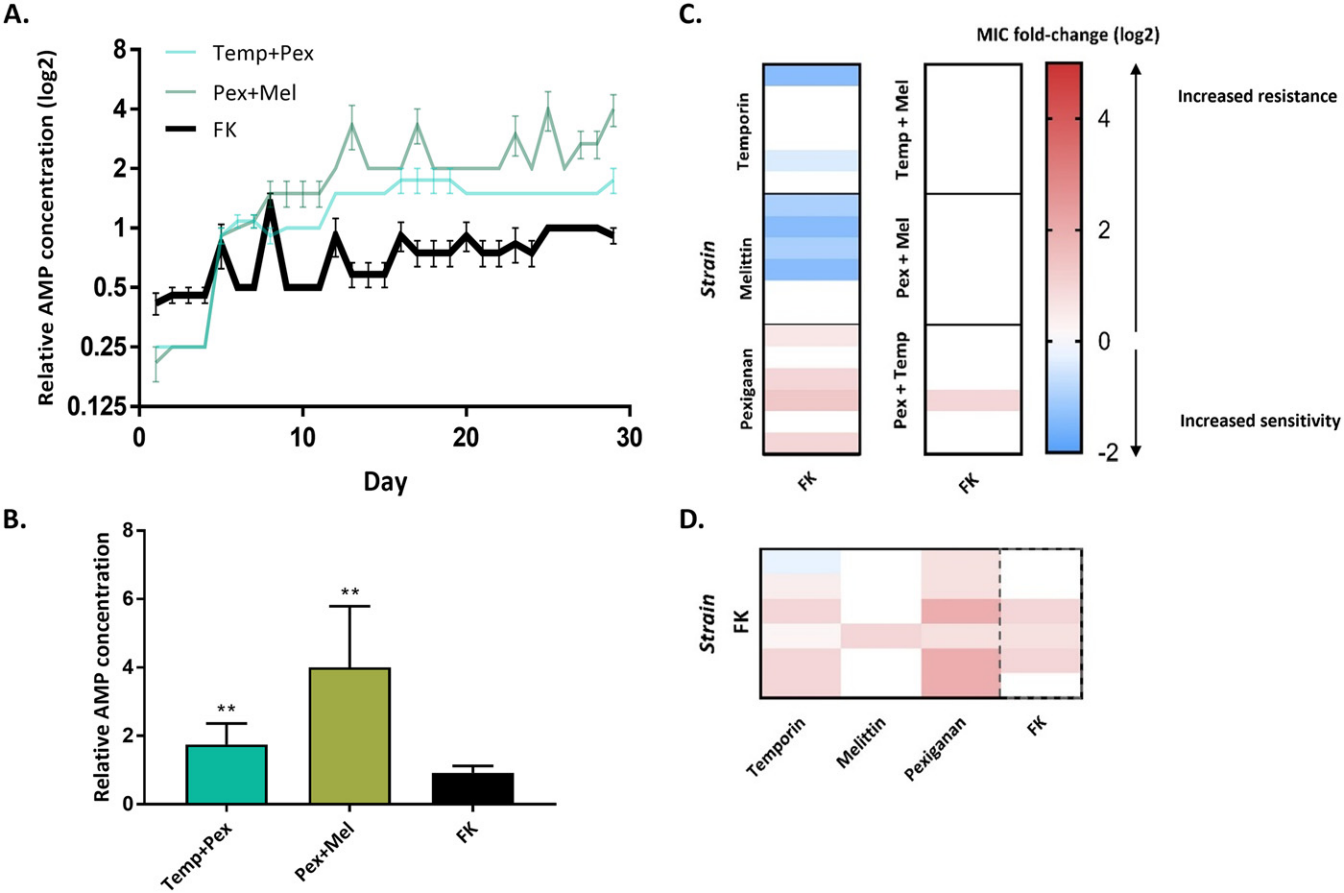
Random-peptides mixture (FK) hinder the evolution of resistance and cross-resistance toward it. (A) Data represent the concentration of AMPs where bacteria grew, normalized to initial MIC (*n* = 6; mean ± SEM; growth defined as an OD_595_ of >0.3. Each graph shows the concentrations of AMPs through the evolution. FK, random peptide mixture. (B) Comparison of the relative AMP concentration after 29 transfers (*n* = 6; mean + SEM). *, *P* < 0.05; **, *P* < 0.01 (Mann-Whitney U test with Bonferroni correction). (C) Susceptibility of evolved strains toward FK20. (D) Susceptibility of FK evolved strains toward different AMPs. Data in panels C and D represent the mean of three independent experiments. Dashed lines indicate the MIC toward the AMP that it evolved with (not cross-resistance or sensitivity).

Given that pairs of AMPs hindered resistance evolution, we next tested whether more diverse AMP combinations may delay resistance evolution even further. We explored a novel type of AMPs, random antimicrobial peptide mixtures (RPMs), a combinatorial library of antimicrobial peptides (33). The peptides in the mixture contain only two types of amino acids, one hydrophobic and one cationic, and a defined chain length of 20 amino acids. Thus, the RPM contains more than 1 million sequences (2^20^ optional sequences in the mixture) of peptides that are all composed of the same two amino acids. RPMs were found to be highly effective against a variety of pathogens, even *in vivo* (34). We evolved the bacteria with 20-mer RPM composed of phenylalanine and lysine (FK), which represents a complexed case of peptide combinations. The MIC to this RPM increased only by a factor of 2 throughout the course of our experimental evolution (Fig. 6A and B), a lower increase than that for any of the individual AMP and 2-AMP combinations (see Fig. S4 in the supplemental material).

We further investigated the sensitivity of the strains that evolved with individual AMP to the RPM (FK). Surprisingly, 6 out of 18 strains develop collateral sensitivity, and 9 strains maintained a similar sensitivity (Fig. 6C). Cross-resistance to FK RPM occurred in four out of six pexiganan-evolved strains. Except for pexiganan strains, no cross-resistance evolved toward FK (only 1/30). Low cross-resistance to individual AMPs also occurred in most of the FK-evolved strains (Fig. 6D), though the strains remain sensitive to melittin (except for one strain). Yet, three out of the six strains remained as sensitive as the ancestor (toward FK), as the rest evolved resistance that increased the MIC by 2-fold maximum. In summary, these results indicate that the AMP-resistant strains could be treated using AMP combinations, with the same efficacy as that of the ancestor. Furthermore, development of general resistance toward all AMPs is less likely to occur.

## DISCUSSION

In the current study, we examined the evolution of resistance of the bacterial pathogen S. aureus toward AMP pairs and the factors that affect the rate with which resistance arises.

We first demonstrated that S. aureus can readily evolve resistance toward selected individual AMPs. The resistance that was observed varied between a 2- and 64-fold increase in the MIC values. Interestingly, the lowest resistance evolved toward AMPs that act on the bacterial membrane via the carpet model, i.e., ovispirin and aurein. In contrast, the highest resistance evolved against pore-forming AMPs (pexiganan, melittin, pardaxin), with temporin being an exception. Further, we have observed that a combination of two AMPs can delay the evolution of resistance compared to each individual AMP. These results are consistent with Dobson et al. (18), who performed experimental evolution with a combination of pexiganan and melittin.

To further explore the reason for the low resistance occurrence, we quantified the fitness cost effect. We have shown that the fitness cost of resistance to AMPs differs by strain and that melittin and pexiganan strains had the most impaired growth fitness. The combinations of these two AMPs substantially hindered the evolution of resistance compared to each AMP alone, suggesting that the high cost of resistance to these AMPs can be an explanation for this combination’s effective reduction of resistance evolution. Antibiotic resistance strains that are clinically isolated are frequently found to have similar fitness in the absence of antibiotics, which strengthen the hypothesis that combinations of high-cost resistance AMPs might be effective for preventing resistance occurrence (35).

Moreover, temporin strains evolved collateral sensitivity toward the AMP pexiganan. This result may contribute to the effectiveness of the combination of temporin-pexiganan. The fact that temporin-resistant strains did not show a fitness cost could select for these strains rather than pexiganan-resistant strains and, therefore, become more sensitive to pexiganan. In contrast, temporin and melittin strains both showed cross-resistance toward each other, which might be a reason for this combination to be less efficient. These results are consistent with previous studies that showed that the collateral sensitivity between antibiotics can limit resistance evolution (36, 37). However, Nichol et al. suggested that collateral sensitivity is never universal and that cross-resistance could be developed if a different evolutionary trajectory is taken (38).

Cross-resistance between AMP-resistant strains is raising concerns since many of them have a shared mechanism of action—disruption of the bacterial membrane (39). We found that although our selected AMPs act on the membrane, they are not sharing cross-resistance across all AMPs. In addition, AMP-resistant strains were still sensitive to a combination of AMP (FK). These results suggest that cross-resistance to immune system AMPs may not evolve so readily due to clinical AMP treatments.

Treatment with a combinatorial library of over 1 million AMP sequences resulted in lower levels of resistance than treatments involving AMP pairs. This suggests that cocktails involving multiple AMPs may be more effective in hindering the evolution of resistance. Nevertheless, it is still unclear whether this requires ultradiverse cocktails, such as the random AMP library included in this study, or whether a defined mixture of several AMPs may suffice. Overall, these results emphasize the potential of AMP combinations to hinder resistance evolution and support the rationale of using antimicrobial peptide mixtures rather than individual AMPs.

In summary, we have demonstrated the ability of antimicrobial peptide combinations to delay the evolution of resistance in the pathogen S. aureus. Further research is needed in order to uncover the genetic and mechanistic basis of resistance to AMPs and to test to what extent the trends that we found hold more broadly across other antimicrobials and pathogens.

## MATERIALS AND METHODS

### Bacterial strains and growth conditions.

All experiments were performed with S. aureus JLA513 (40) (*hla-lacZ hla*^+^, derived from SH1000; kindly provided by Jens Rolff), which contains tetracycline resistance. This strain is defined as the ancestor strain. Prior to each experiment, strains were isolated from Mueller-Hinton (MH) (HiMedia) agar plates, and individual colonies were picked and grown in MH broth overnight in 37°C. All bacterial cells used in this study were stored in 25% glycerol at −80°C.

### Synthesis of antimicrobial peptides.

Six different AMPs with three different modes of action were selected for the experimental evolution assay (see Table S1 in the supplemental material). All AMPs were synthesized using 9-fluorenylmethoxy carbonyl (Fmoc) solid-phase peptide synthesis (SPPS) method using peptide synthesizer (Liberty Blue; CEM, USA). Upon synthesis completion, peptides were cleaved from the resin (95% trifluoroacetic acid [TFA], 2.5% water, and 2.5% triisopropylsilane [TIPS]), resuspended in double distilled water (DDW), frozen, and lyophilized. Subsequently, the crude peptide was dissolved in dimethyl sulfoxide (DMSO) and purified using semipreparative reversed-phase high-performance liquid chromatography (RP-HPLC) (see Fig. S5 in the supplemental material), while matrix-assisted laser desorption ionization–time of flight mass spectrometry (MALDI-TOF-MS) was utilized for verification of the peptide mass and purity. Random peptide mixtures (RPM) containing phenylalanine and lysine (FK20) were synthesized as previously described (41).

### MIC determination.

MIC values were determined using a standard protocol (42). Briefly, S. aureus cells were grown overnight in MH broth at 37°C and 200 rpm. Subsequently, cells were diluted 1:100 in MH broth and grown until reaching an optical density at 595 nm (OD_595_) of 0.1. Then, 100 μL of 5 × 10^5^ CFU/mL was inoculated into each well in 96-well plates that contained a serial dilution of AMPs. Each plate contained 3 replicates of each AMP. MIC value were defined as the lowest concentration at which there is inhibition of bacterial growth by at least 90%. Fold change of MIC in evolved strains was divided by the ancestor MIC.

Cross-resistance and collateral sensitivity were evaluated by the same method as MIC assay. Each strain was exposed to different AMPs and the AMP that it evolved with as control. Ancestor MICs were determined as well in each experiment. Each experiment was repeated at least twice independently.

### Experimental evolution procedure.

Prior to evolution with AMPs, an S. aureus JLA513 colony was transferred from an MH agar plate into 5 mL MH broth in a 50-mL tube and incubated overnight in 37°C and 200 rpm. Subsequently, this starter culture was diluted by 20-fold into a 1.5-mL Eppendorf tube containing 850 μL MH broth to maintain the same headspace ratio as in the experimental evolution procedure and incubated under the same conditions (37°C and 200 rpm) overnight. These dilutions by 20-fold were repeated for 7 transfers in order to habituate the bacteria to the experimental conditions. Our experimental evolution procedure was designed to exert selective pressure yet to avoid extinction of lines. Therefore, each line was exposed to 4 concentrations of AMP according to its MIC as follows: 1.5×, 1×, 0.5×, and 0.25× (Fig. 1; see also Table S1). Experiments were performed in 96-well plates, and each AMP had 6 parallel lines (same ancestor). In the AMP combination treatments, the effective ratio between the AMPs was 1:1, as the MIC contained 0.5× MIC of each. In each plate, 8 wells with bacteria only (no AMPs) were used as a positive control. Four wells with medium only serve as negative control to indicate contaminations. Six lines that evolved with rifampicin were used as positive control for resistance evolution, as S. aureus evolves resistance toward it readily (43). Every day, 10 μL of the previous plate was replicated into 190 μL of fresh medium and AMPs. Every 4 days, bacteria from the highest concentration of AMP were selected and transferred into 4 concentrations in the new plate. MIC was doubled when growth was observed in 4 out of 6 lines in MIC or higher. Growth was defined as an OD of >0.3. The experimental evolution was carried out for 29 transfers, which are approximately 122 generations. Before every selection or MIC increment, samples were taken to make glycerol stocks (25%) and preserved in −80°C to avoid line extinction. Spot plating was performed on MH agar containing 5 μg/mL tetracycline to indicate growth before selection.

### Fitness cost of evolved strains.

Bacterial cells were grown overnight in MH broth and then diluted to an OD_595_ of 0.1/100. Two hundred microliters of each strain’s culture was transferred into 96-well plates. Each strain had 3 repeats in the same plate. Optical density (OD_595_) was measured every 15 min for 24 h using Epoch 2 microplate reader (BioTek). Lag time, *V*_max_, and *t* at *V*_max_ were calculated using the plate reader software (Gen5) and normalized to the ancestor strain’s values. The area under the curve was calculated by OD_595_ measurements until stationary phase (14 h).

### AMPs interaction assessment using checkerboard assay.

Before proceeding to evolution with combinations of AMPs, the interaction between AMPs has been assessed. In order to evaluate these interactions, checkerboard assays were performed as previously described (42). Briefly, 100 μL of 5 × 10^5^ CFU/mL log-phase S. aureus cells was added into 96-well plates with different concentrations of two AMPs. The plates were then incubated for 24 h in 37°C, and bacterial growth was determined by measuring optical density (OD_595_) using a plate reader. The checkerboard assay results were used to calculate the fractional inhibitory concentration index (FICI) according to the following equation:

$${\mathrm{FICI}}_{\mathrm{AB}}=\frac{{\mathrm{MIC}}_{\text{A}}^{\mathrm{Comb}}}{{\mathrm{MIC}}_{\text{A}}^{\mathrm{Alone}}}+\frac{{\mathrm{MIC}}_{\text{B}}^{\mathrm{Comb}}}{{\mathrm{MIC}}_{\text{B}}^{\mathrm{Alone}}}$$

where ${\mathrm{MIC}}_{\text{A}}^{\mathrm{Comb}}$ is the MIC of peptide A when combined with peptide B, and ${\mathrm{MIC}}_{\text{A}}^{\mathrm{Alone}}$ is the MIC peptide A alone without the presence of compound B. Finally, the overall interactions between peptides were quantified in two methods as follows: minimal FIC and average FIC. The minimal FIC is the lowest concentration (FIC) where bacteria were inhibited by at least 80%. In the average FIC method, we calculated the FIC for each concentration that bacteria were inhibited by at least 80%, and then the average FIC for all values was calculated. Each experiment was performed in duplicate. The results represent two independent experiments.
