# Supplementary material for: Antimicrobial Peptide Combination Can Hinder Resistance Evolution
Source: Microbiol Spectr. 2022 Jul 13;10(4):e00973-22. doi: 10.1128/spectrum.00973-22 (PMC9430149; doi:10.1128/spectrum.00973-22)
Supplement: Supplemental file 1 — Supplemental material. Download spectrum.00973-22-s0001.pdf, PDF file, 0.2 MB [file spectrum.00973-22-s0001.pdf]

Supporting information

Table S1. Antimicrobial peptides sequences and antimicrobial activity against ancestral strain

AMP	*Sequence*	*MIC (µg/ml)*	*Purity (%)*
Ovispirin	KNLRRIIRKIIHIIKKYG	50	>97
Aurein 1.2	GLFDIIKKIAESF	50	>92
Melittin	GIGAVLKVLTTGLPALISWIKRKRQQ	6.25	>99
Pexiganan	GIGKFLKKAKKFGKAFVKILKK	12.5	>94
Temporin A	FLPLIGRVLSGIL	6.25	>97
Pardaxin	GFFALIPKIISSPLFKTLLSAVGSALSSSGGQE	100	>90

Table S2. Minimal inhibitory concentrations (MIC) of the no-peptide selected strain (NPSA)

	MIC (µg/ml)		
AMP	**Ancestor**	**Exp.1**	**Exp.2**
Temporin	6.25	6.25	6.25
Melittin	6.25	6.25	6.25
Pexiganan	12.5	12.5	12.5
Pardaxin	100	200	-
Ovispirin	50	25	-
Aurein	50	50	-
FK	100	100	100

Table S3. AMP interactions using checkboard assay

Combination	***Average FICi***	***Min FICi***
Pexiganan + Melittin	indifference (1.18)	indifference (1)
Temporin + Melittin	indifference (1.78)	indifference (1)
Temporin + Pexiganan	indifference (1.03)	partial synergy (0.75)

Table S4. Statistical test and analysis summary

Figure	**Test**	**n**	**Samples**	**Test value (U/ Z)**	**Adjusted p-values**	**Summary**
Fig 1B	Kruskal-Wallis test	6 (nmel=5)	All strains	29.29 (Kruskal-Wallis)	<0.0001	**
Fig 1B	Dunn's multiple comparison	6,5	Ovispirin vs. Melittin		0.0006	**
		6,6	Ovispirin vs. Temporin A		>0.9999	ns
		6,6	Ovispirin vs. Aurein 1.2		>0.9999	ns
		6,6	Ovispirin vs. Pexiganan		0.7442	ns
		6,6	Ovispirin vs. Pardaxin		0.0122	*
		5,6	Melittin vs. Temporin A		0.028	*
		5,6	Melittin vs. Aurein 1.2		0.0006	**
		5,6	Melittin vs. Pexiganan		0.3864	ns
		5,6	Melittin vs. Pardaxin		>0.9999	ns
		6,6	Temporin A vs. Aurein 1.2		>0.9999	ns
		6,6	Temporin A vs. Pexiganan		>0.9999	ns
		6,6	Temporin A vs. Pardaxin		0.3135	ns
		6,6	Aurein 1.2 vs. Pexiganan		0.7442	ns
		6,6	Aurein 1.2 vs. Pardaxin		0.0122	*
		6,6	Pexiganan vs. Pardaxin		>0.9999	ns
Fig S1-B	MWU with Bonferroni correction	6,6	Temporin vs. Melittin	0	0.0066	**
		6,6	Pexiganan vs. Melittin	0	0.0066	**
		6,6	Pexiganan vs. Temporin	3.5	0.0456	*
Fig 2 D-F	MWU with Bonferroni correction	6,6	Temp+Mel vs. Temp	2	0.0216	*
		6,6	Temp+Mel vs. Mel	3	0.026	*
		6,6	Temp+Pex vs. Temp	0	0.013	*
		6,6	Temp+Pex vs. Pex	2	0.0044	**

		6,6	Mel+Pex vs. Mel	0	0.0044	**
		6,6	Mel+Pex vs. Pex	0	0.0044	**
Fig 3	MWU with Bonferroni correction	6,6	Mel vs. Pex+Mel	0	0.0044	**
		6,6	Mel vs. Temp+Mel	2	0.0174	*
		6,6	Pex vs. Pex+Mel	2.5	0.0216	*
		6,6	Pex vs. Pex+Temp	0.5	0.0086	**
		6,6	Temp vs. Mel+Temp	7	0.1212	ns
		6,6	Temp vs. Pex+Temp	0	0.0044	**
Fig 4A	Dunn's multiple comparison	6,6	Ancestor vs. Temporin		0.6585	ns
Lagtime		6,5	Ancestor vs. Melittin		0.0078	**
		6,5	Ancestor vs. Pexiganan		0.3323	ns
		6,6	Ancestor vs. NPSA		>0.9999	ns
Fig 4B	Dunn's multiple comparison	6,6	Ancestor vs. Temporin		>0.9999	ns
Vmax		6,5	Ancestor vs. Melittin		0.0057	**
		6,5	Ancestor vs. Pexiganan		0.0004	**
		6,6	Ancestor vs. NPSA		0.0873	ns
Fig S2	Dunn's multiple comparison	6,6	Ancestor vs. Temporin		>0.9999	ns
T at Vmax		6,5	Ancestor vs. Melittin		0.0118	*
		6,5	Ancestor vs. Pexiganan		0.4823	ns
		6,6	Ancestor vs. NPSA		0.7347	ns
Fig S3	Dunn's multiple comparison	6,6	Ancestor vs. Temporin		>0.9999	ns
AUC		6,5	Ancestor vs. Melittin		0.05	*
		6,5	Ancestor vs. Pexiganan		0.0095	**
		6,6	Ancestor vs. NPSA		>0.9999	ns
Fig 5	Fisher's exact test	12,12	Pexiganan vs. Temporin		0.0003	**
Fig 6B	MWU with Bonferroni correction	6,6	FK vs. Pex + Temp	0	0.0044	**
		6,6	FK vs. Pex + Mel	0	0.0044	**

Statistical analysis performed using GraphPad Prism 7.02. * p<0.05, ** p<0.01

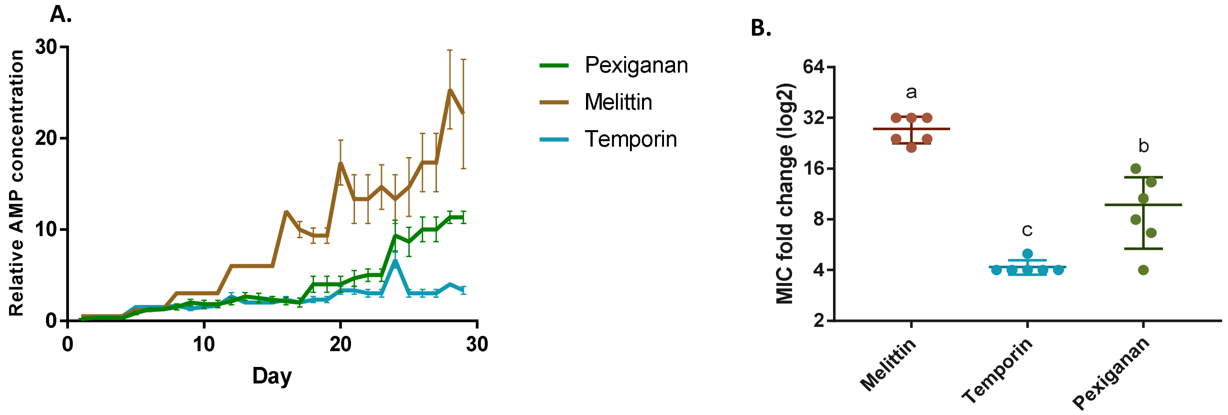

Fig S1. Combination of AMPs can hinder the evolution of resistance. (A) Relative concentration of individual AMPs through the experimental evolution (combination experiment). (B) Resistance of the individual-AMP evolved strains (MWU with Bonferroni correction, a-c represent significant differences between groups). (n=6, Growth defined as OD_{595} >0.3). The MIC determined towards the AMP it evolved with. Each bar represents the mean of six lines + SEM. * p<0.05, ** p<0.01 (Mann-Whitney U test with Bonferroni correction).

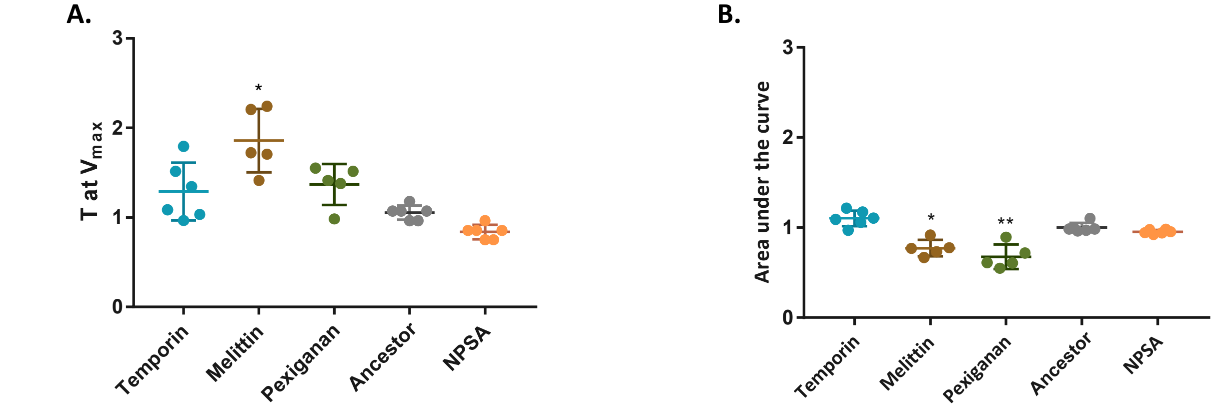

Fig S2. Fitness cost of evolved strains. Determination of fitness cost performed by growing the bacteria in the absence of AMPs. OD_{595} was measured every 15 min through 24 hours. (A) Time to achieve maximal growth rate (V_{max}). (B) Area under the curve. Values were calculated by plate reader software (Gen 5 and normalized to ancestor strain values). Each dot represents mean of triplicate, bars represent mean ± SD ($n_{melittin,\ pexiganan}$=5, Dunn's multiple comparison refed to ancestor strain, * p< 0.05, ** p< 0.01). NPSA- evolved strain without AMPs. The results represent three independent experiments.

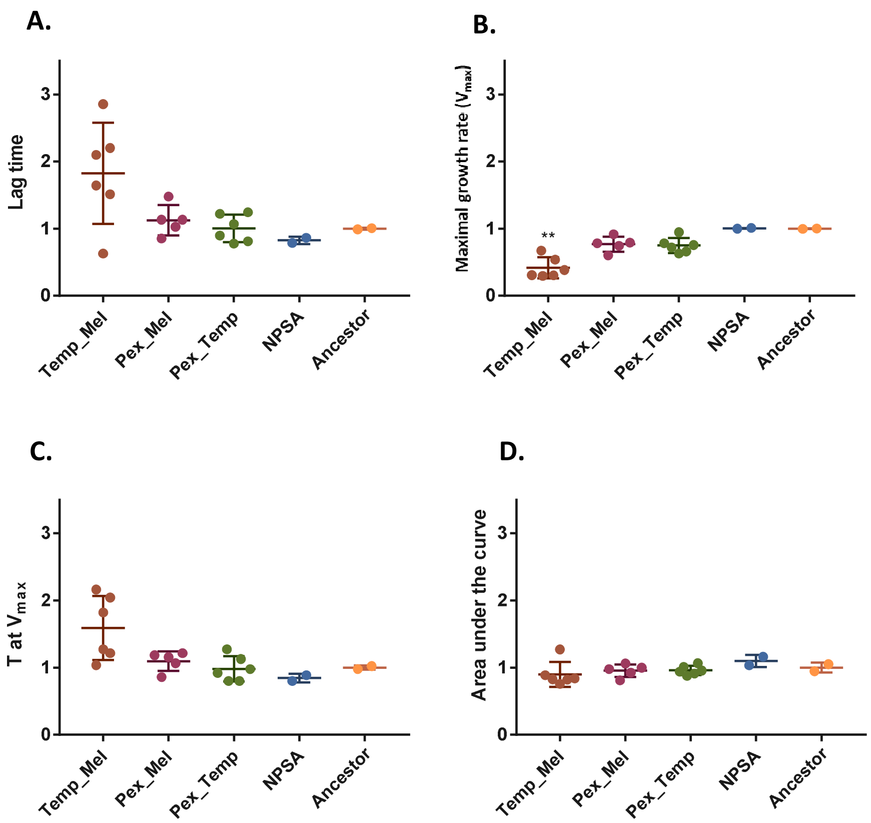

Fig S3. Fitness cost of AMP-combinations evolved strains. Determination of fitness cost performed by growing the bacteria in the absence of AMPs. OD_{595} was measured every 15 min through 24 hours. (A) Lag time, (B) maximal growth rate (Vmax), (C) time at Vmax, (D) area under the curves. A-C values were calculated by plate reader software (Gen 5 and normalized to ancestor strain values). Each dot represents mean of triplicate, bars represent mean ± SD (n=6, Dunn's multiple comparison refed to ancestor strain, ** p< 0.01). NPSA- evolved strain without AMPs. The results represent three independent experiments.

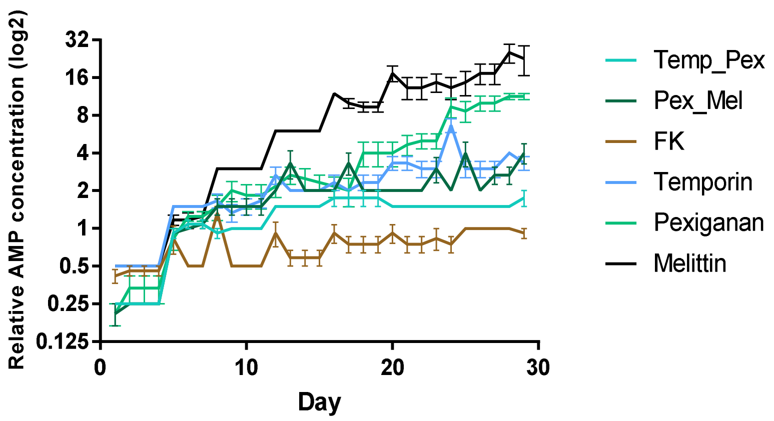

Fig S4. Random peptides mixture composed of phenylalanine and lysine (FK) delays the evolution of resistance. Relative AMP concentration through the evolution. The concentrations are normalized to the initial MIC. Bars represent the mean + SEM.

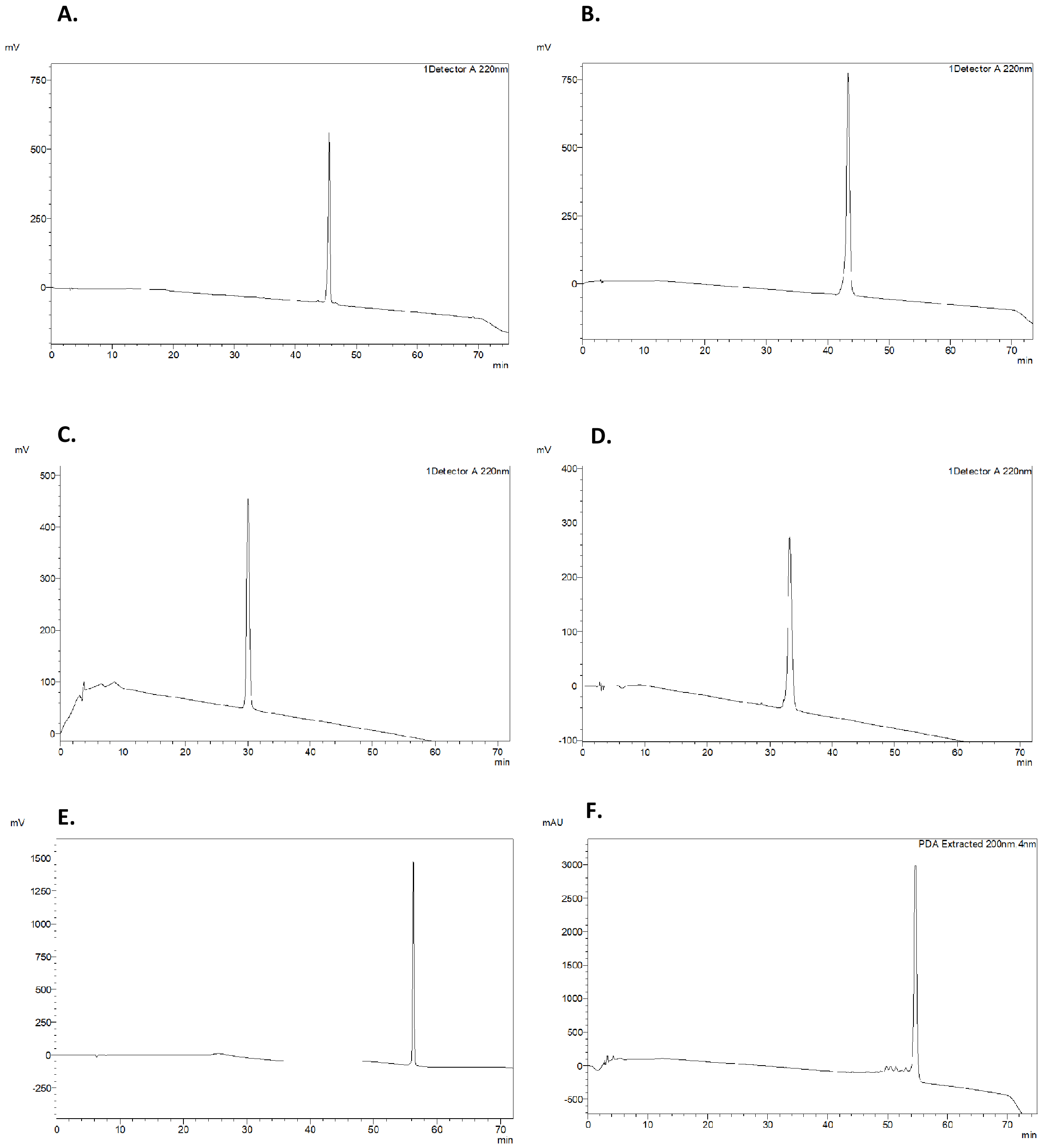

Fig S5. HPLC chromatograms of purified AMPs. The purification chromatograms of AMPs using reverse-phase high-performance liquid chromatography (HPLC). (A) Temporin, (B) melittin, (C) pexiganan, (D) ovispirin, (E) aurein, (F) pardaxin. The purified AMP dissolved in 20% acetonitrile, then injected to a gradient of 5 to 65 percent acetonitrile with increment of 0.5% in a minute.
